# Effects of Pulsed Electric Field Treatment on Compression Properties and Solutes Diffusion Behaviors of *Jerusalem artichoke*

**DOI:** 10.3390/molecules24030559

**Published:** 2019-02-03

**Authors:** Zhenzhou Zhu, Rui Zhang, Nabil Grimi, Eugene Vorobiev

**Affiliations:** 1 College of Food Science and Engineering, Wuhan Polytechnic University, Wuhan 430023, China; zhenzhouzhu@126.com; 2 Laboratoire Transformations Intégrées de la Matière Renouvelable (UTC/ESCOM, EA 4297 TIMR), Centre de recherche Royallieu, Université de Technologie de Compiègne, CS 60319, 60203 Compiègne Cedex, France; nabil.grimi@utc.fr (N.G.); eugene.vorobiev@utc.fr (E.V.)

**Keywords:** pulsed electric field, compression kinetic, diffusion, *Jerusalem artichoke*, inulin

## Abstract

*Jerusalem artichoke* is widely used as raw material for industrial production of inulin. Pressing (compression) and diffusion are two effective technologies for bio-compounds’ recovery from plants. In this work, pulsed electric field (PEF) treatment at 400, 600, and 800 V/cm during 100 ms was applied to facilitate juice and solutes recovery from *Jerusalem artichoke*. The application of PEF led to electroporation of cell membranes and enhanced the tissue compression/juice expression and solutes diffusion. The consolidation coefficient (calculated by application of semi-empirical model) of PEF treated sample at 800 V/cm was 6.50 × 10^−7^ m^2^/s, which is significantly higher than that of untreated sample (5.02 × 10^−9^ m^2^/s) and close to that of freeze-thawed sample. Diffusion experiments with PEF treated samples were carried out at 25, 50, and 75 °C. A PEF treatment of *Jerusalem artichoke* at 800 V/cm led to a similar diffusion behavior at 25 °C, compared to diffusion behavior obtained from untreated sample at 75 °C.

## 1. Introduction

Inulin is a kind of fructan, which is widely used in food products for fat replacement and calorie reduction [1]. Although, inulin is presented in various plants, the raw materials for industrial production are chicory root and *Jerusalem artichoke* [2,3]. Chicory root, which is mainly planted in Europe (especially Belgium), is considered to be the most adapted plant for the industrial production of inulin [4]. Due to climate reasons, *Jerusalem artichoke* is widely planted in China and is the most important material for inulin production in China [3]. 

Conventionally, hot water extraction at 70–80 °C during 1.5–2 h is applied for inulin extraction [5,6]. The high temperature and prolonged duration ensure the extraction yield, however, leads to penetration of impurities (for instance proteins and colorants) into the extract due to the breakage of cellular membranes and cell walls. Consequently, the purification process needs complex steps, such as clarifying, filtration, and decoloration [7,8], implying important waste water emission and high energy consumption.

To search for alternative technologies for inulin extraction with higher purity and less energy consumption, various investigations have been carried out [2,3,9]. The application of pulsed electric fields (PEF) with very short duration (generally from several microseconds to several milliseconds) was effective for compounds’ extraction from plants, thanks to the formation of cell membrane pores temporarily or permanently [10,11,12,13,14,15]. For chicory root, PEF pre-treatment (600 V/cm, 50 ms) gave an inulin extraction yield at 60 °C that was comparable with that obtained at 80 °C without PEF treatment, and with an even higher purity [2,13]. However, to our best knowledge, PEF assisted inulin extraction from *Jerusalem artichoke* was not investigated until now.

Apart from aqueous extraction, pressing is other unit operation to separate liquid containing compounds from the solid-liquid matrix by mechanical compression. The application of PEF to assist the compression process has also been carried out. Mhemdi et al. [16] investigated combined pressing-diffusion technology for sugar beets pretreated by PEF. The results indicated that with PEF assisted pressing, sugar beet slices were better exhausted, permitting a reduction of the sucrose loss in pulp from 1.2% to 0.8%, and extracted more concentrated sucrose that was less colored and purer than that conventionally extracted by diffusion. A detailed investigation about the effect of PEF treatment on the pressing behavior of sugar beet tissue was also reported [17]. The calculated consolidation coefficient (filtration diffusivity) revealed that a PEF treatment of 600 V/cm during 10 ms induced better compression than that without PEF treatment. Grimi et al. [18] explained that the PEF treatment led to the removal of the entrapped air by the liquid released from the electroporated cells, thus modifying the compressibility of the tissue. Until now, the PEF assisted pressing of *Jerusalem artichoke* was not investigated.

**Figure 1:**
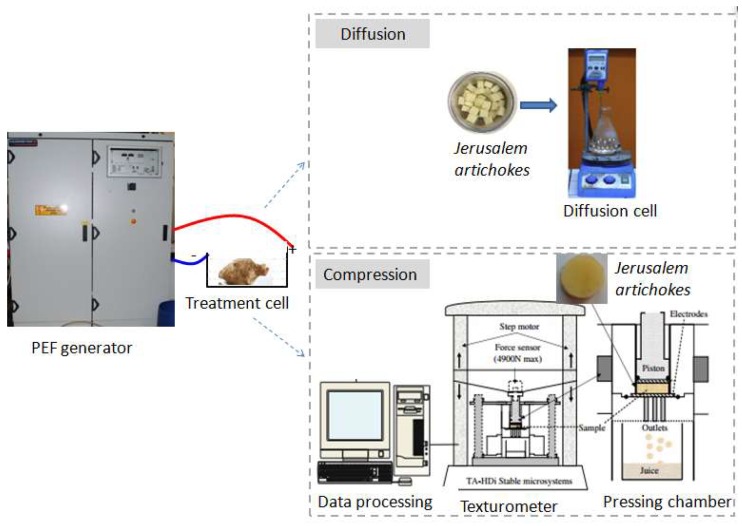
Set-up used for pulsed electric fields (PEF) treatment, texture analysis, and diffusion.

The aim of this work is to study the effect of PEF treatment (with differing intensity) on the compression kinetics and filtration diffusivity of the *Jerusalem artichoke* (Figure 1), and to evaluate its influence on inulin extraction. For this purpose, PEF treatments at 400, 600, and 800 V/cm were applied to assist the compression and diffusion process. Consolidation coefficients characterizing the filtration diffusivity and diffusion behavior are investigated. 

## 2. Results and Discussion

### 2.1. Deformation Curves and Data Computing

**Figure 2:**
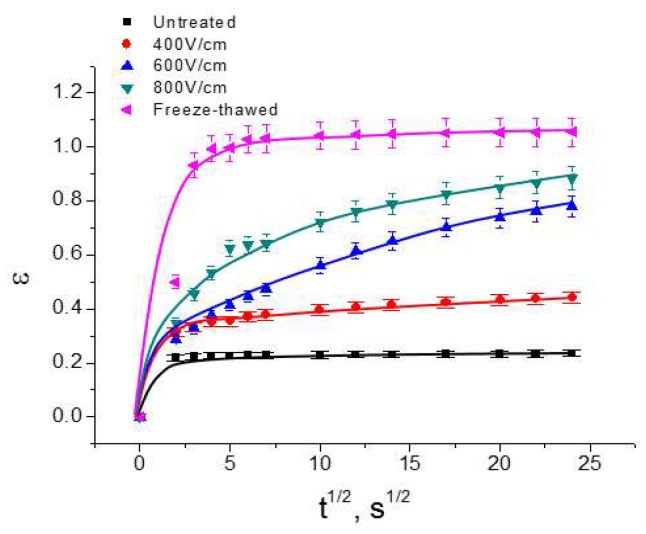
Deformation, *ε*, vs. *t*^1/2^ for untreated, PEF treated, and freeze-thawed samples.

Figure 2 represents the relative deformation, *ɛ* vs. *t*^1/2^, for untreated, PEF treated, and freeze-thawed *Jerusalem artichokes* samples. Since the loading time required for attaining the fixed constant-pressure (4 bar) was very short, the loading of samples is assumed to be quasi-instantaneous. For untreated samples, a quasi-immediate elastic deformation was obtained in almost 5 s. The relative deformation reached 0.22 in 5 s. It should also be noted that the deformation curve presents two obvious steps: almost linear at the beginning of the compression (first step), and a slow deformation until the end of the compression (second step). Comparatively, PEF treatment and freeze-thawed treatment led to more important deformation of samples. For example, the final relative deformation reached 0.44, 0.78, and 0.88 for PEF treated samples under 400, 600, and 800 V/cm, respectively. In fact, previous studies have shown that PEF treatment at 400, 600, and 800 V/cm could lead to different degrees of tissue electroporation, thus resulting in differing compression behavior [19]. The freeze-thawed treatment resulted in the highest final relative deformation.

**Figure 3:**
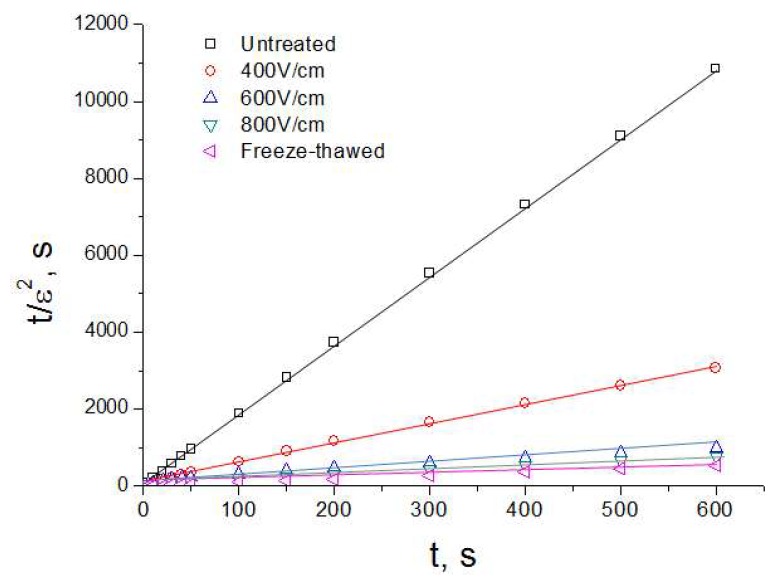
$\frac{t}{\varepsilon^{2}}$ vs. *t* for the untreated, PEF treated, and freeze-thawed samples.

**Figure 4:**
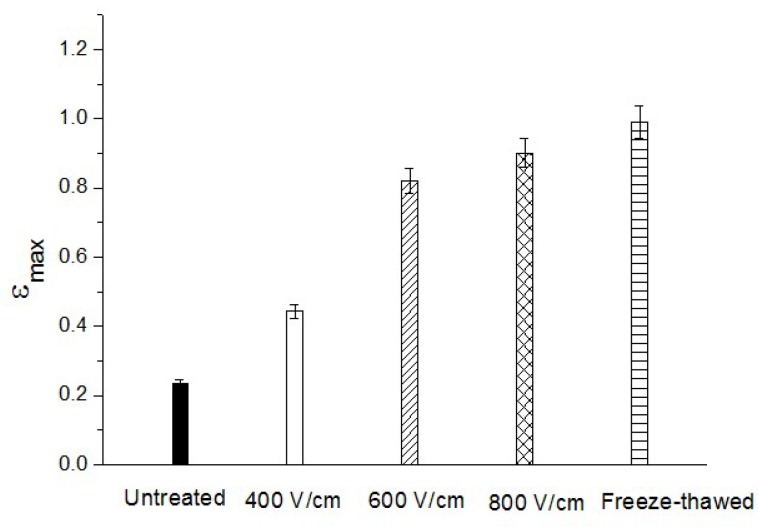
$\varepsilon_{\infty }$ for the untreated, PEF treated, and freeze-thawed samples.

Figure 3 represents the deformation curve in coordinates $\frac{t}{\varepsilon^{2}}$ vs. *t* according to Equation (4). This good linear correlation between $\frac{t}{\varepsilon^{2}}$ and *t* validate the applicability of the above equations for compression tests with *Jerusalem artichoke* tissue, and was used to find the slope *c*, and to calculate the value of $\varepsilon_{\infty }$. The values of $\varepsilon_{\infty }$ obtained from Figure 3 for different treatments are presented in Figure 4. The maximal relative deformation varied according to the treatments. PEF treatment significantly increased the maximal relative deformation of samples due to the better electroporation of tissue. At the highest values of PEF intensity, the increase of $\varepsilon_{\infty }$ became less pronounced. For example, the value of $\varepsilon_{\infty }$ increased from 0.44 to 0.82 when the electric field strength increased from *E* = 400 to *E* = 600 V/cm, while $\varepsilon_{\infty }$ reached 0.90 at *E* = 800 V/cm. At this value of *E*, $\varepsilon_{\infty }$ was close to that obtained for freeze-thawed sample. However, it should be noted that the $\varepsilon_{\infty }$ value also depends on the applied pressure [20]. 

**Figure 5:**
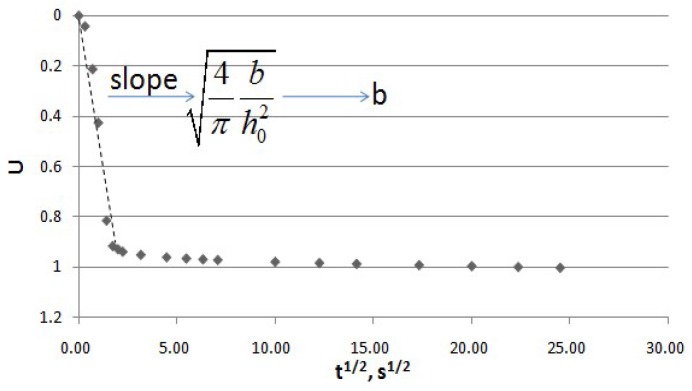
*U* vs. *t*^1/2^ for the determination of *b*.

**Figure 6:**
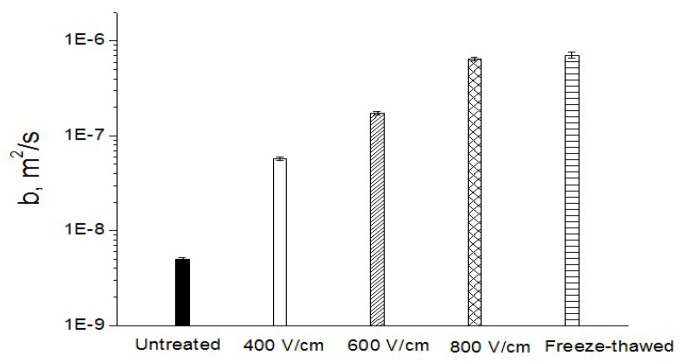
Coefficient for the untreated, PEF treated, and freeze-thawed samples.

Curves of *U* vs. *t*^1/2^ allow the calculation of the consolidation coefficient, *b*. One example of this is presented in Figure 5. The slope of the initial part of the *U* vs. *t*^1/2^ relationship corresponds to Equation (3), from which the value of *b* can be determined. Figure 6 shows the values of the consolidation coefficient, *b*, for the untreated and PEF treated samples. More intensive PEF treatment led to the higher values of *b*. Closed values of *b* for the sample treated by PEF at *E* = 800 V/cm and for the freeze-thawed sample indicate the efficiency of PEF for *Jerusalem artichoke* tissue. The value of the maximal consolidation coefficient of *Jerusalem artichoke* tissue seems comparable with that of sugar beet, which was reported previously [17,18] (4 × 10^−7^ m^2^/s), implying the similar compressibility of these two plant materials.

### 2.2. Diffusion

**Figure 7:**
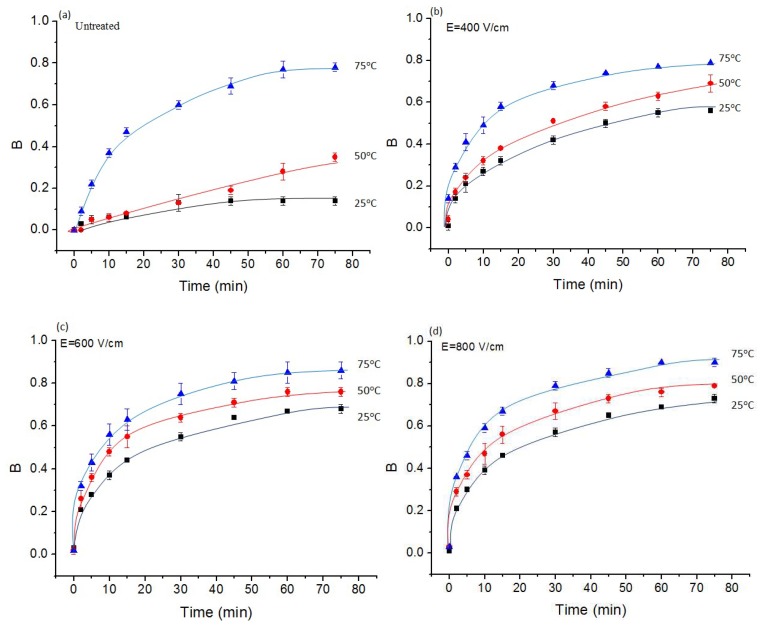

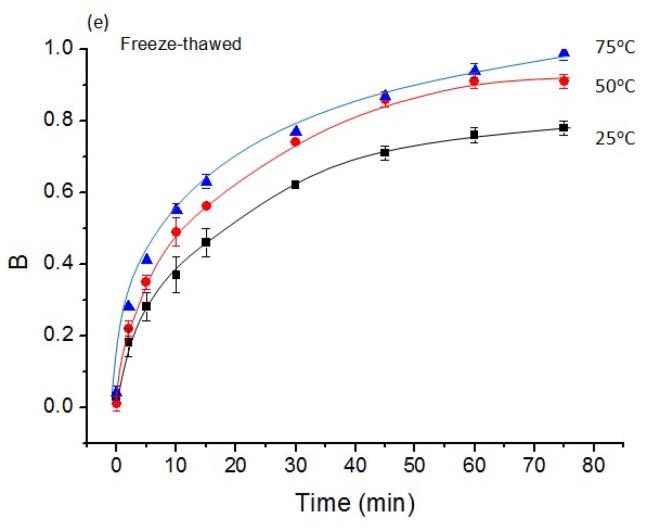
Normalized Brix function, *B*, during aqueous extraction from untreated (**a**), PEF treated at 400 V/cm (**b**), 600 V/cm (**c**), 800 V/cm (**d**), and freeze-thawed (**e**) *Jerusalem artichokes.*

Diffusion experiments at 25, 50, and 75 °C were carried out to evaluate the effect of the pre-treatment and diffusion temperature on soluble matter extraction. Figure 7 shows the solute extraction kinetics from *Jerusalem artichokes* untreated, PEF treated (*E* = 400–1000 V/cm), and freeze-thawed. The results are presented in the form:

(1) $$B=\frac{{}^{0}Brix-{}^{0}Brix_{i}}{{}^{0}Brix_{f}-{}^{0}Brix_{i}}$$

where *^0^Brix_i_* and *^0^Brix_f_* are the initial and final soluble matter contents, respectively, and *B* is the normalized function of *^0^Brix*. The value of *^0^Brix_i_* was determined immediately after placing *Jerusalem artichokes* into the diffusion cell, and *^0^Brix_f_* = 4.0 ± 0.2 was determined after 2 h of extraction at 80 °C. 

A previous study [2] showed a good linear correlation (*R^2^* = 0.97) between the inulin (measured by HPLC) and the soluble matter content (°*Brix*) in the extracted juice from chicory roots. Here, we suppose that the total solutes’ extraction kinetics from *Jerusalem artichokes* corresponds roughly to the variation of the inulin content during the extraction experiment. The data presented in Figure 7 show that more rapid diffusion kinetics can be achieved at higher diffusion temperatures. The maximal value of *B* for the untreated sample was 0.78, corresponding to the diffusion temperature of 75 °C. However, the same value of *B* was achieved by freeze-thawing at 25 °C, indicating that the diffusion temperature might be reduced after pretreatments. Figure 7 b–d revealed that PEF treatment accelerated the solute diffusion, especially at low diffusion temperatures (25 °C). However, for the diffusion at 75 °C, the positive influence of PEF treatment was lower. These results revealed that the denaturation of plant tissue can be caused by both thermal and electrical treatment. To reach a satisfactory solute diffusion, an effective PEF pre-treatment can lead to less thermal treatment requirements, therefore, reducing the diffusion temperature. 

## 3. Materials and Methods 

### 3.1. Materials

Commercial *Jerusalem artichokes* were selected as the raw material for investigation. Samples of good and uniform quality were purchased from the supermarket (Compiegne, France). The samples were stored at 4 °C until required. The moisture content of *Jerusalem artichokes* was within 75%–80%. Freeze-thawed sample was prepared at −18 °C for 24 h, followed by thawing of the sample at ambient temperature. The rectangle-shaped samples, which were 5 mm high, 10 mm long, and 10 mm wide, were prepared for diffusion experiments. Cylinder-shaped samples (diameter *d* = 25 mm and height *h* = 10 mm) were prepared for compression experiments using a texture analyzer (model TA-XT Plus, Stable Microsystems, Surrey, England).

### 3.2. PEF Treatment 

PEF treatment was carried out using a PEF generator, 5 kV-1 kA (Hazemeyer, Saint-Quentin, France) (Figure 1). The whole sample of *Jerusalem artichokes* was used in the PEF treatment process. The samples were placed in the PEF treatment cell. The PEF treatment cell was composed of two plane electrodes (120 mm × 205 mm) and filled with distilled water media to obtain a good contact. The distance between the two electrodes was fixed at 6 cm, which corresponded to a treatment volume of 1.48 × 10^−3^ m^3^. The value of the electric field strength, *E*, was evaluated as *E* = *U/d* (V/cm), where *U* (V) was the applied voltage and *d* (cm) was the distance between the two electrodes. The PEF generator provided pulses of a near-rectangular shape. In the present study, 10 trains, where 100 pulses with a duration of *t_i_* = 100 µs in each, was applied. The pause between individual trains was Δ*t* = 10 s. The total time of the PEF treatment was 100 ms. The choice of PEF treatment time was based on a previous study of PEF assisted solute extraction from chicory roots [21]. The electrical field strength was varied from 400–800 V/cm.

### 3.3. Diffusion Experiments

The instrument for the extraction of soluble matter from *Jerusalem artichokes* was a Fisher Scientific laboratory apparatus(San Jose, CA, USA) equipped with a magnetic stirrer (-Thermo Fisher, San Jose, CA, USA) and digital thermo regulator (10–1300 min^−1^,Thermal Fisher, San Jose, CA, USA) (Figure 1). The extraction experiments were conducted in a glass round-bottomed flask with rectangle-shaped samples (80 g) and distilled water. The distilled water was preheated at the desired temperature (25 °C, 50 °C, and 75 °C, respectively), then rectangle-shaped samples were placed into the extraction cell. In every extraction test, the liquid to solid radio was fixed at 4 (*w/w*). The speed of agitation was fixed at 150 rpm. 

To measure the soluble matter content (°Brix, %), 1mL of the solution was taken at 0, 2, 5, 10, 15, 30, 45, 60, and 75 min. The round-bottomed flask was covered by aluminum foil during experiments to avoid any evaporation. The soluble matter content (°Brix, %) was measured by a digital Atago refractometer (PR-101, Atago, 50 Tokyo, Japan). 

### 3.4. Compression Test

The compression test was carried out with a texture analyzer (model TA-XT Plus, Stable Microsystems, Surrey, England) equipped with a 4905 N load cell (Figure 1). It consists of a pressing piston and a cylinder-shaped force senor (diameter of 25 mm), both made of stainless steel. The cylinder-shaped samples (diameter of 25 mm and height of 10mm) were placed over the cylinder-shaped force senor. A stainless-steel pressing piston with a force of 200 N (which is the maximum force offered by the applied equipment, and the pressure corresponds to 4 bar), moving at a speed of 1 mm/s, was used to evaluate the deformation of the sample. The thickness (*h*) of the sample during compression was recorded by the equipped computational software (Texture Expert Exceed). The deformation was defined as *ε* = 1 − *h_t_/h*_0_, where *h_t_* and *h*_0_ are the current and initial thickness of the specimen, respectively. Then, the displacement-time curve was analyzed. For good experimental practice, all tests were run on the same day the samples were processed.

### 3.5. Statistic Analysis

All the tests were repeated at least three times. Data were expressed as the mean ± standard deviation (SD). 

### 3.6. Solid-Liquid Expression Model

Although the mechanism of solid–liquid expression from plant materials is still under investigation, the filtration-consolidation theory is currently widely adopted for simulation of the mechanical expression of cellular materials [17,22,23,24]. According to this theory, with the assumptions that the plant tissue is composed of compressible porous materials and an average filtration diffusivity exists during the consolidation process, the following equation was developed, presenting the relationship between consolidation ratio (*U*) and expression time (*t*, s) [18,25,26]:

(2) $$U=\frac{\Delta h}{\Delta h_{\infty }}=\frac{\varepsilon }{\varepsilon_{\infty }}=\frac{{\left[\frac{4}{\pi }\frac{b}{h_{0}^{2}}t\right]}^{0.5}}{{\left[1+{\left(\frac{4}{\pi }\frac{b}{h_{0}^{2}}t\right)}^{\upsilon }\right]}^{0.5/\upsilon }}$$

where Δ*h = h_0_ − h*, Δ*h_∞_ = h*_0_ *− h_∞_*, *h*_0_ (m) and *h* (m) are the initial and actual sample thickness, $\varepsilon =\frac{\Delta h}{\Delta h_{0}}$ and $\varepsilon_{\infty }=\frac{\Delta h_{\infty }}{\Delta h_{0}}$ are the actual and infinite relative deformation, respectively, *b* (m^2^/s) is the consolidation coefficient characterizing filtration diffusivity, and υ is the consolidation behavior index.

From Equation (2), the following relation is proposed [25] for the small values of *t*:

(3) $$U={\left[\frac{4}{\pi }\frac{b}{h_{0}^{2}}t\right]}^{0.5}$$

Then, the value of *b* could be obtained from the slope of the plot, *U* vs. *t*^1/2^.

The infinitive relative deformation, *ε*, can be obtained from the relation [27,28]:

(4) $$\frac{t}{\varepsilon^{2}}=a+ct$$

where *a* and *c* are constants. According to Equation (4), the value of *c* can be found from the slope of the plot, $\frac{t}{\varepsilon^{2}}$ vs. *t*. From Equation4, at infinite time, *ct* ≫ *a*, thus ε∞=1c. After obtaining the value of $\varepsilon_{\infty }$, the value of *U* can be found, then the consolidation coefficient, *b,* can be determined from the slope of the curve, *U* vs. *t*^1/2^ (Equation (3)).

## 4. Conclusions

PEF pre-treatment at 400, 600, and 800 V/cm was applied to enhance the tissue compression and solutes’ extraction from *Jerusalem artichokes*. The relative deformation was approximately 0.9 after 800 V/cm PEF treatment during 100 ms, close to that obtained by freeze-thawing. Consolidation coefficients of untreated, PEF treated, and freeze-thawed samples were determined by application of the semi-empirical model. The consolidation coefficient of the PEF treated sample at 800 V/cm was 6.50 × 10^−7^ m^2^/s, significantly higher than that of the untreated sample (5.02 × 10^−9^ m^2^/s), and close to that of the freeze-thawed sample. Diffusion experiments led to the determination of solute diffusivity from the untreated and PEF treated tissue. The electric field intensity (*E*) showed great influence on the diffusion behavior. Both the compression and diffusion tests evidenced that a PEF treatment of 800 V/cm was sufficient for electroporation of *Jerusalem artichokes*. Nearly the same diffusion behavior was attained for the untreated sample at 75 °C and the PEF treated (800 V/cm) sample at 25 °C. This gives an interesting perspective for the new technologies of “cold” extraction from *Jerusalem artichokes*.
